# Numerical Analysis of the Influence of Porosity and Pore Geometry on Functionality of Scaffolds Designated for Orthopedic Regenerative Medicine

**DOI:** 10.3390/ma14010109

**Published:** 2020-12-29

**Authors:** Piotr Prochor, Anita Gryko

**Affiliations:** Faculty of Mechanical Engineering, Institute of Biomedical Engineering, Bialystok University of Technology, Wiejska 45C Street, 15-351 Bialystok, Poland; grykoanita@gmail.com

**Keywords:** scaffold, pores, geometry, orthopedics, numerical methods

## Abstract

Background: Scaffolds are vital for orthopedic regenerative medicine. Therefore, comprehensive studies evaluating their functionality with consideration of variable parameters are needed. The research aim was to evaluate pore geometry and scaffold porosity influence on first, cell culture efficiency in a perfusion bioreactor and second, osteogenic cell diffusion after its implantation. Methods: For the studies, five pore geometries were selected (triangular prism with a rounded and a flat profile, cube, octagonal prism, sphere) and seven porosities (up to 80%), on the basis of which 70 models were created for finite element analyses. First, scaffolds were placed inside a flow channel to estimate growth medium velocity and wall shear stress. Secondly, scaffolds were placed in a bone to evaluate osteogenic cell diffusion. Results: In terms of fluid minimal velocity (0.005 m/s) and maximal wall shear stress (100 mPa), only cubic and octagonal pores with 30% porosity and spherical pores with 20% porosity fulfilled the requirements. Spherical pores had the highest osteogenic cell diffusion efficiency for porosities up to 30%. For higher porosities, the octagonal prism’s pores gave the best results up to 80%, where no differences were noted. Conclusions: The data obtained allows for the appropriate selection of pore geometry and scaffold porosity for orthopedic regenerative medicine.

## 1. Introduction

The scaffold is a porous structure whose key role is to provide temporary or permanent mechanical integrity at the site of tissue damage until it is repaired or regenerated and its normal biomechanical function is restored [1,2,3]. In this case, the possibility of recovering the mechanical integrity of a bone can be understood as enabling the load transfer through bone that is divided into at least two segments due to several reasons. Currently, scaffolds are also used in orthopedic regenerative medicine to rebuild a bone fragment that has been excised due to neoplastic changes, crushed by excessive mechanical loads, or has been subject to other factors causing the need to remove it and create bone discontinuity [4,5,6,7,8,9,10]. A properly designed structure of the scaffold allows the restoration of human efficiency to a degree that is impossible to achieve with the use of conventional treatment methods [4,11].

Scaffolds are structures that are in continuous development. Currently, many researchers from various scientific environments (i.e., materials science, bioengineering, manufacturing engineering, computational science, and engineering) are trying to further develop this specific type of implant with the use of various experimental and numerical methods. The main goal is to determine the optimal construction and material parameters that allow the highest possible functionality of the scaffold to be obtained.

Melchels et al. used experimental and numerical methods to investigate the effect of two selected scaffold structures on the cell distribution [12]. They used scaffolds synthesized by a ring opening polymerization of photo-polymerizable poly (d,l-lactide) (PDLLA) and further treatment in methacrylic anhydride. The first variant was an isotropic model with a nearly constant pore size of 412 ± 13 µm and a porosity of 62 ± 1%, whereas the second one had a variable pore size in the range of 250–500 µm and porosity of 35% to 85%. Computational fluid dynamics (CFD) models presented uniform distribution of flow velocity and wall shear stress (WSS) for isotropic architecture and variable flow velocity and wall shear stress for the second analyzed structure. The highest cell density was correlated with the scaffold areas where pores were large and fluid velocity as well as wall shear stresses were highest. This means that cell deposition depends mainly on the local stress values, which are dependent on the pore size and type [12].

Zhao et al. used CFD to examine the functionality of scaffolds with highly irregular pore geometry, created by micro-computed tomography (micro-CT) scanning of a silk fibroin (SF) scaffold [13]. The simulation was carried out in a simulated channel of a perfusion bioreactor, where the analyzed scaffold geometry was placed. By assigning appropriate material properties and boundary conditions, they determined the value of the wall shear stress and the velocity of the growth medium inside the scaffold. The stresses obtained on the walls of the scaffolds ranged from 0 mPa to 50 mPa, whereas for velocity the values were 0 mm/s to 2 mm/s [13].

Byrne et al. presented a fully three-dimensional approach to the computer simulation of tissue differentiation and bone regeneration in a scaffold with regular pore geometry as a function of porosity, Young’s modulus, and material dissolution rate (without considering any specific material type) under low and high load conditions [14]. They used a mechanoregulation algorithm, which allowed the evaluation of the tissue differentiation process both in terms of the dominant biophysical stimulus and the number of precursor cells. Their research showed that initially, osteogenic cells were practically absent in the granulation tissue filling the pores of the scaffold. As the simulation progressed, osteogenic cells proliferated to form small clusters that eventually differentiated according to the intensity of the mechanical stimulus, in the end creating a tissue with new material properties. The applied load produced a stimulus that promoted osteogenesis, leading to bone formation in the center of the scaffold and soft tissue on its external sides, where high stress concentrations occurred. Their simulations confirmed that all three analyzed variables had an effect on the amount of bone formed, but not in an intuitive way. In a low-load environment, higher porosity and higher stiffness, as well as the average dissolution rate, give the greatest amount of bone, whereas in an environment with high load, the dissolution rate should be lower, otherwise the scaffold would collapse. However, at lower porosity, higher dissolution rates can be used [14].

In general, studies can be divided into two main groups: studies with the use of experimental or numerical methods. All of them present the importance of scaffolds not only in orthopedic regenerative medicine to repair long bone defects [15,16], but also to restore osteochondral [17,18], maxillofacial [19,20], or even spinal [21] damage. Moreover, a properly designed scaffold can also be a drug delivery structure [22].

Despite the existence of a significant amount of research presenting comprehensive observations, it is still impossible to clearly define the optimal parameters of scaffolds designated for orthopedic regenerative medicine. This suggests that there is a strong need for further research to understand as many factors as possible that have an impact on their functionality. For this reason, the aim of the study was to fill the current gap with the analyses of the influence of porosity and pore geometry on the functionality of selected scaffolds.

## 2. Materials and Methods

Numerical studies were conducted within modules of ANSYS Workbench v19.2 software: fluid flow (Fluent), which is a module for CFD calculations, and transient structural, which allows for the evaluation of the process of osteogenic cell diffusion in the granulation tissue formed at the fracture site. Computer Aided Design (CAD) models of analyzed scaffolds were designed in SolidWorks 2019 software. Scaffold models were created with the consideration of two variables: pore geometry and porosity.

**Figure 1:**
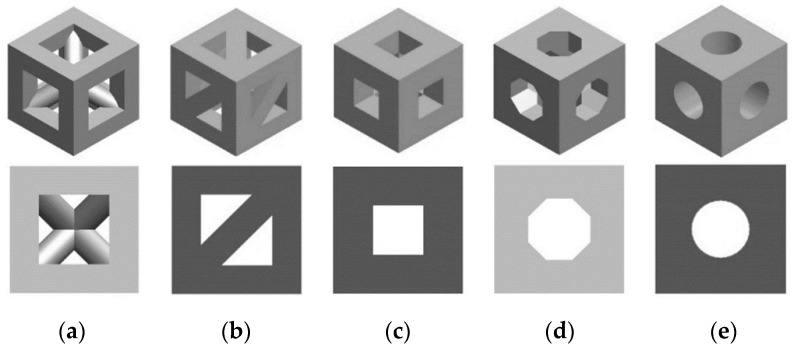
Analyzed geometries of pores on the example of a unit cell: (**a**) triangular prism with a rounded profile, (**b**) triangular prism with a flat profile, (**c**) cube, (**d**) octagonal prism, and (**e**) sphere.

In the first variable, i.e., the geometry of pores, five shapes were selected: a triangular prism with a rounded (Figure 1a) and a flat profile (Figure 1b), a cube (Figure 1c), an octagonal prism (Figure 1d), and a sphere (Figure 1e). The pore geometries used in conducted analyses were selected on the basis the data of the reference presenting commonly used and evaluated shapes of scaffolds [23,24]. It was decided to consider simple geometries that in practice can be used to create nearly all more complex shapes. Moreover, the use of pores with defined geometry allowed for a reduction in the influence of notches, randomly created with the use of complex, randomized geometries. 

**Figure 2:**
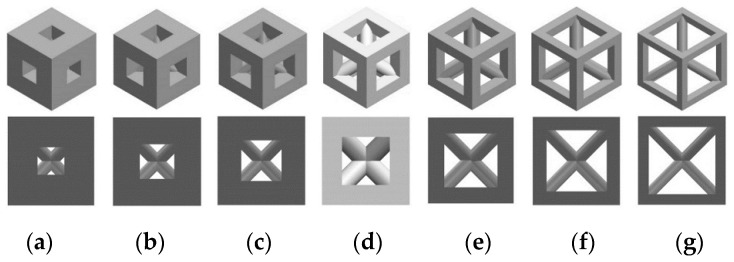
Analyzed porosities of scaffolds on the example of a selected unit cell (triangular prism with a rounded profile): (**a**) 20%, (**b**) 30%, (**c**) 40%, (**d**) 45%, (**e**) 60%, (**f**) 70%, and (**g**) 80%.

The seconds variable, i.e., porosity, was obtained by changing the size of the pores, keeping their number constant in the scaffold volume. Despite the changes in the geometry of the pore, the proportion of the material to empty space was the same for all analyzed structures in case of selected porosities. Seven types of porosity were chosen: 20%, 30%, 40%, 45%, 60%, 70%, and 80%, which so far have been used in experimental conditions and computational simulations [25,26]. In regenerative medicine, porosities higher than 40% are often considered. However, with the increase in porosity, mechanical properties of a scaffold decrease. For this reason, lower porosities must be also evaluated in order to estimate the functionality of scaffolds that can provide better support in terms of high load transfer that occurs in long bones. The consideration of porosities used in both practical and numerical cases was crucial, as the research goal was to estimate the efficiency of most variables used in current studies. The influence of porosity on the shape of a cell unit, on the example of a triangular prism with a rounded profile, is shown in Figure 2.

### 2.1. Growth Medium Velocity and Wall Shear Stress inside Scaffolds

**Figure 3:**
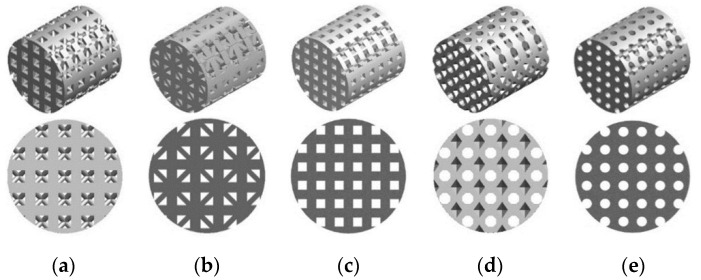
Exemplary CAD models prepared for analyses of growth medium velocity and wall shear stress inside scaffolds: (**a**) triangular prism with a rounded profile, (**b**) triangular prism with a flat profile, (**c**) cube, (**d**) octagonal prism, and (**e**) sphere.

In the first part of the study the process of a cell culture within a scaffold placed in a perfusion bioreactor was evaluated in order to estimate growth medium velocity and wall shear stress inside scaffolds. It was simulated by modelling a bioreactor channel (60 mm length and 10 mm diameter) where the cylindrical scaffolds, created from previously described cell units, with a height of 10 mm and diameter of 10 mm, were placed in the middle of it. A total of 35 scaffold structures were made and tested in terms of a flow of growth medium through the scaffold. Figure 3 presents an example of evaluated scaffold geometries with a porosity of 45% and all shapes tested.

**Figure 4:**
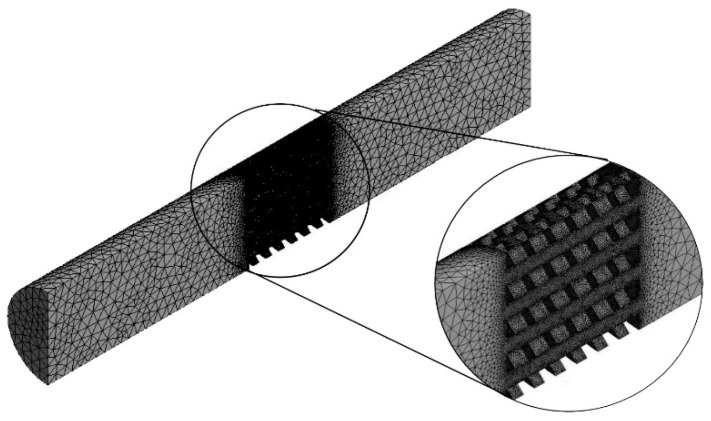
Finite element model obtained after discretization with the consideration of a 5% mesh refinement test (example on a scaffold with cubic pores and a porosity of 45%).

Channel models were adjusted by subtracting the scaffold geometry from the volume. Subsequently, mesh was created with the use of 4-node finite elements and a 5% convergence test for both growth medium velocity and WSS. The number of finite elements in the models was approximately 1,300,000 ± 200,000. Figure 4 presents an example of a model obtained after discretization.

Appropriate parameters were set during the research in order to simulate the cell culture process in the channel of the perfusion bioreactor. The density and dynamic viscosity of the growth medium as well as its initial velocity and temperature were used (Table 1). A non-slip feature was assumed for the scaffold wall. For this reason, the consideration of scaffold material was not required as the analyzed results were not dependent upon the scaffold’s material properties. This assumption is widely used in the analyses of fluid flow through scaffolds in a perfusion bioreactor [27].

As stated before, the velocity of the growth medium and WSS were calculated during simulations with the use of a double precision, pressure-based solver. It was proven that low velocities can disturb the appropriate washing away of metabolic waste or even prevent osteogenic differentiation [29,30,31,32]. At the same time, a too-high velocity can lead to apoptosis of cells [29,30,33]. Achieving appropriate velocity can enhance the deposition of the mineralized matrix, which positively affects the cell culture process [34,35,36,37]. In the case of WSS, it stimulates the growth of cells; however, if it is too high, it can detach cells from the scaffold’s internal walls [13,28]. For this reason, the considered obtained values of the analyzed parameters create an upper and lower threshold that can be used to evaluate the functionality of any scaffold. The upper limit is the maximum allowable value of WSS, whereas the lower limit is the minimal velocity of the growth medium. 

**Figure 5:**
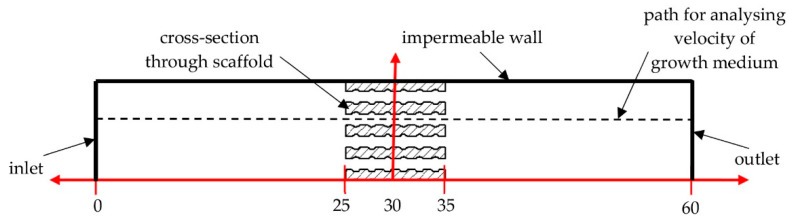
Scheme of the analyzed models with boundary conditions and a path for analyzing the growth medium velocity.

Although the maximal values of WSS were taken from all internal walls of the scaffold, the velocity results were taken from the path passing through the pores exactly in the center of the scaffolds (Figure 5). An impermeable wall was modeled to include the area of growth medium flow limited by the inner diameter of the bioreactor channel. No slip as the shear condition as well as no wall motion were considered.

### 2.2. Diffusion of Osteogenic Cells to Granulation Tissue inside Scaffolds

In the second part of the study, a case of the direct use of a scaffold to restore long bone function, without an initial cell culturing process in the perfusion bioreactor, was analyzed. Although the use of a bioreactor can hasten restoration possibilities, a clean scaffold can be used immediately after its manufacturing, which can shorten hospitalization. For this reason, both methods are commonly used in clinical practice.

**Figure 6:**
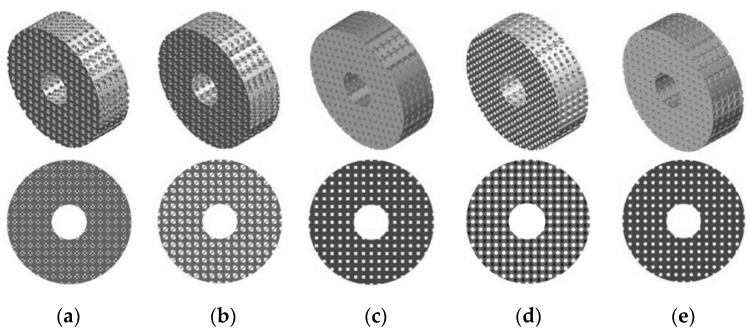
Exemplary CAD models prepared for analyses of the diffusion of osteogenic cells to granulation tissue inside scaffolds: (**a**) triangular prism with a rounded profile, (**b**) triangular prism with a flat profile, (**c**) cube, (**d**) octagonal prism, and (**e**) sphere.

In this part, a simulation of the diffusion of osteogenic cells in the granulation tissue occurred between bone fragments at the site of the scaffold placement. Determination of the diffusion efficiency allowed for the estimation of when the scaffold could be subjected to loading. For the research purposes, scaffolds with an outer diameter of 32 mm, an inner diameter of 10 mm, and a height of 10 mm were modeled. The outside diameter was intended to simulate the outside diameter of the long bone diaphysis, in this case the femur, whereas the inner diameter simulated the medullary cavity, a consideration that also allowed for the facilitation of the propagation of osteogenic cells into the pores of the scaffold. Besides the enhancement of the osteogenic cell diffusion process, the inner hole must be considered to improve further load transfer after obtaining secondary osteointegration (similar to the one of natural bone). As in the previous part of the presented study, 35 scaffold models were again prepared. Figure 6 presents an example of the prepared models of all analyzed shapes with a porosity of 45%.

**Figure 7:**
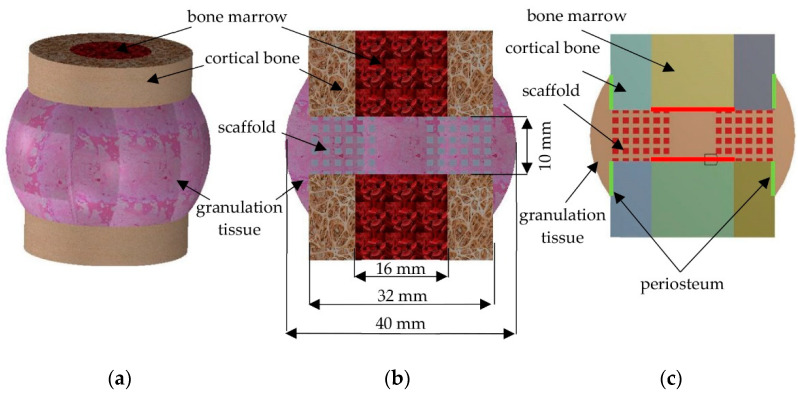
Components of a model for diffusion studies: (**a**) isometric view of three-dimensional model, (**b**) a cross-section through a three-dimensional model, and (**c**) two-dimensional model used for analyses.

The scaffold is placed between bone fragments to restore continuity of the bone and its basic function of transferring loads [38]. For this reason, in order to simulate the bone tissue environment and its healing process, all analyzed scaffolds were placed in a simplified bone model along with a bone marrow and a granulation tissue (Figure 7). The anatomical dimensions of the femur diaphysis and the resulting granulation tissue were used to create the model [39]. Due to computational complicity, three-dimensional models were simplified to two-dimensional ones, created by a cross-section through the center of the model. Periosteum and bone marrow were considered as the source of osteogenic cells. The models’ simplification was possible as the considered bone-implant geometries were characterized by appropriate symmetries. This, as well as the previously described features, allowed for an analysis of the two-dimensional models, as was done in related research [40,41,42].

**Figure 8:**
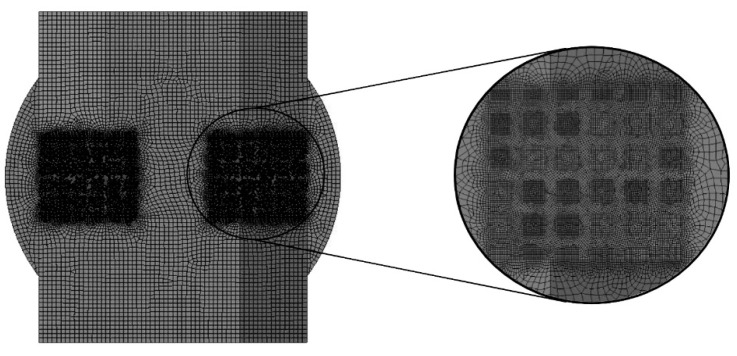
Model for analyses of osteogenic cell diffusion after discretization (example on a scaffold with cubic pores and a porosity of 45%).

Mesh was created with the use of 8-node Plane223 elements and with a consideration of a 5% convergence test for the analyzed results (content of osteogenic cells in granulation tissue). The applied elements allowed the diffusive degree of freedom in the nodes to be unlocked. Moreover, the node-sharing method was included to bond each part of tested models, which consisted of approximately 35,000 ± 20,000 elements (Figure 8).

In simulations of the process of osteogenic cell diffusion, the Fick’s law was applied, which is presented in the equation [43]:

J = −D × (δØ/δx),(1)

where J is the diffusion flux [kg/m^2^ × s], D is the diffusion coefficient [m^2^/s], Ø is the concentration [kg/m^3^], and x is the distance from the source of the diffusing substance [m].

To include Fick’s law, a subroutine was created with the use of Ansys Parametric Design Language (APDL). A single iteration was considered as equal to one day of osteogenic cell diffusion. This allowed the changes in content of osteogenic cells in granulation tissue to be estimated over days of the healing process. The adapted simulation method of the diffusion process simplifies phenomena occurring in real conditions. However, the literature presents that its use allows the data that approximates the actual diffusion of osteogenic cells within the callus to be obtained [44]. The material properties used during analyses of this phenomenon are presented in Table 2. Titanium alloy (Ti6Al4V) was considered as the material the scaffolds were made of as its biocompatibility with appropriate mechanical strength can fulfil the requirements of scaffolds designated for orthopedic regenerative medicine, especially in terms of recovering long bone damage as presented in the data of the references [45,46]. Evaluated structures can be manufactured with currently know support-free 3D-printing methods, such as selective laser sintering (SLS), which makes it possible to use them in experimental conditions [47,48].

## 3. Results

### 3.1. The Influence of Pores Geometry and Scaffold Porosity on Growth Medium Velocity and Wall Shear Stress inside Scaffolds

**Figure 9:**
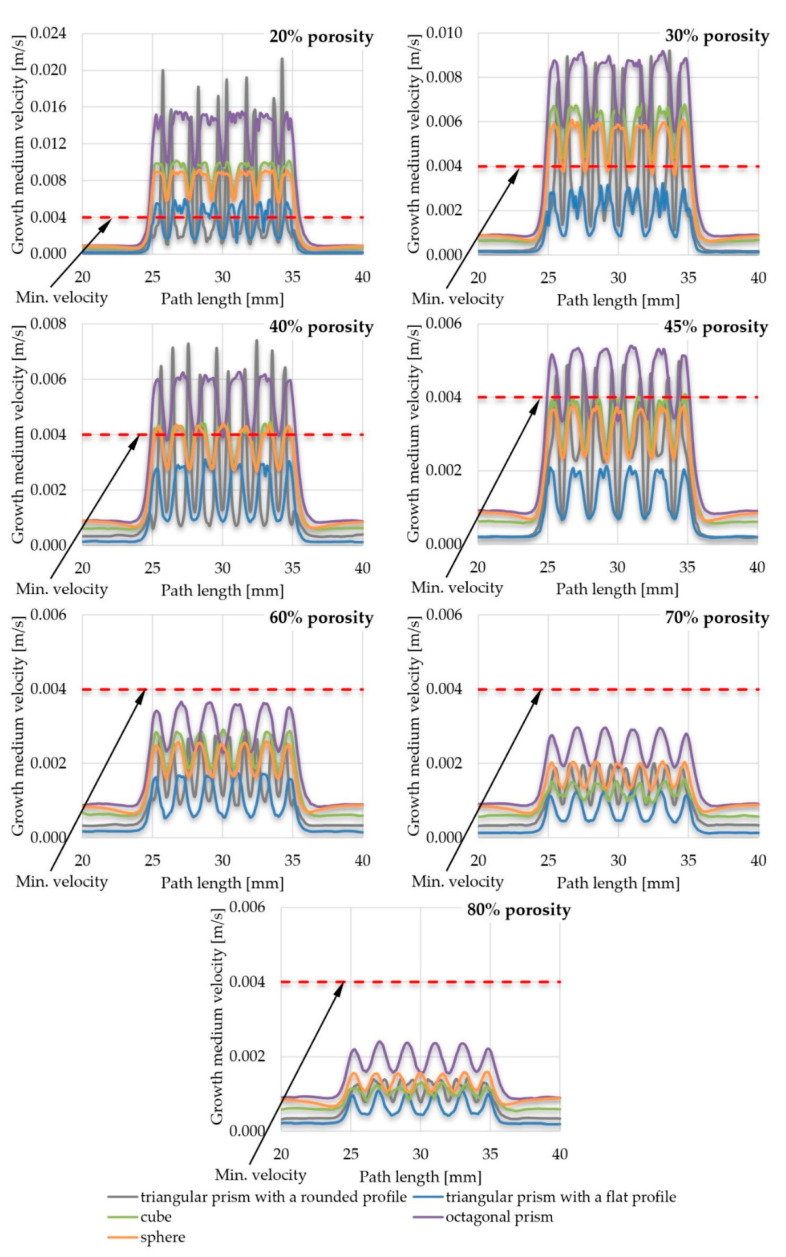
The influence of pore geometry on the growth medium velocity inside a scaffold.

**Figure 10:**
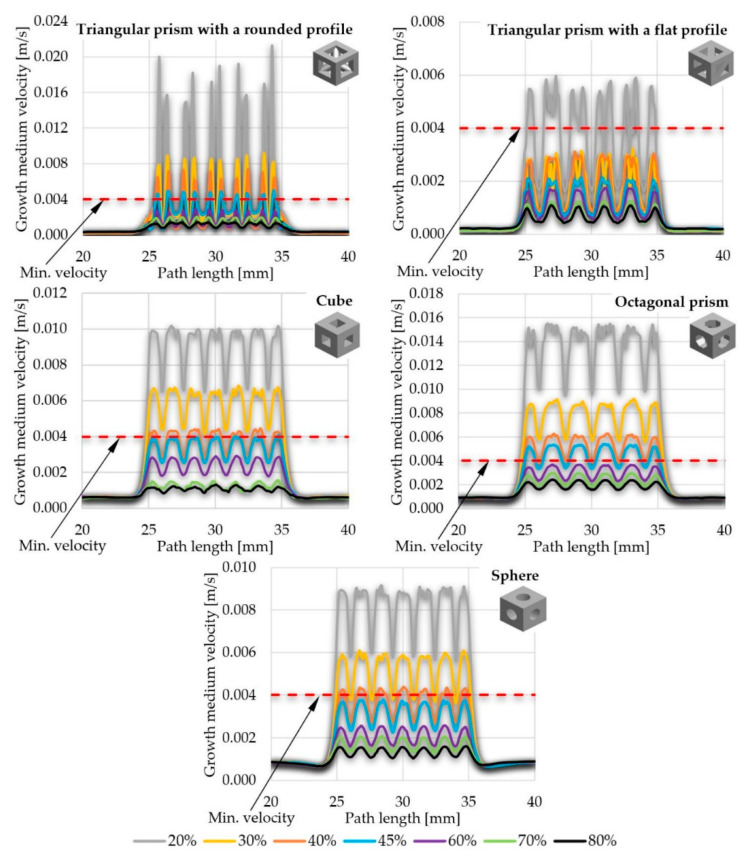
The influence of scaffold porosity on the growth medium velocity inside a scaffold.

The results of the velocity of the growth medium through the scaffold pores are presented in the form of comparative graphs. In order to improve the readability of the results, various combinations were created, namely, a comparison of the efficiency of pore geometry for selected scaffold porosities (Figure 9) and a comparison of the efficiency of scaffold porosity for selected pore geometries (Figure 10). All graphs are shown for the path length (Figure 5) in the range of x ϵ (20; 40). The value of the minimum velocity of growth medium, which ensures the proper exchange of nutrients between it and the cultured cells, was set to 0.004 m/s, which was determined on the basis of the data of the reference [12]. Lower values suggest that the appropriate exchange of nutrients would be disturbed and, at the same time, waste products could not be removed from the inside of the scaffold. A minimal velocity of 0.004 m/s was a lower threshold for further evaluation of the influence of selected parameters on the scaffold efficiency. To present the results more comprehensively, additional views of streamlines of the growth medium velocity inside scaffolds are presented in supplementary materials (Figures S1–S5).

**Figure 11:**
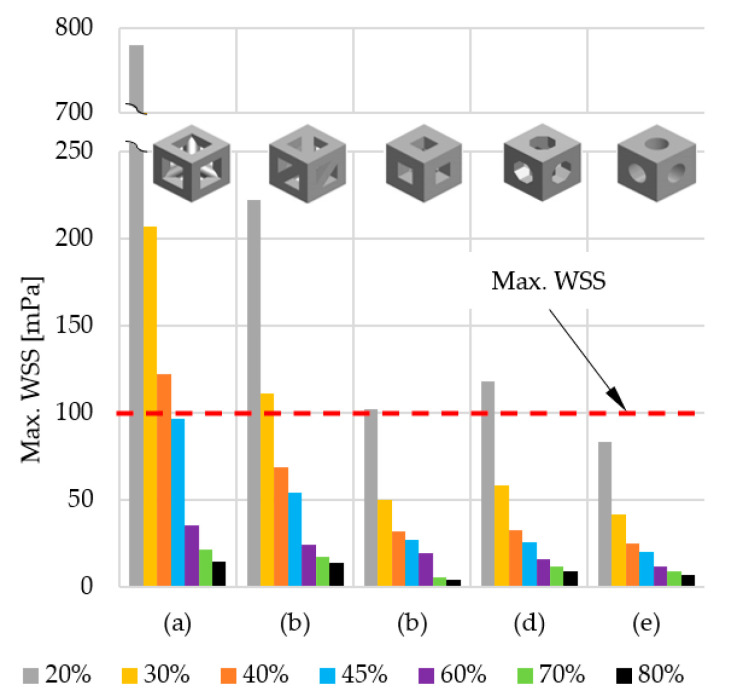
The influence of scaffold porosity and the geometry of pores on the maximal WSS value generated inside scaffold: (**a**) triangular prism with a rounded profile, (**b**) triangular prism with a flat profile, (**c**) cube, (**d**) octagonal prism, and (**e**) sphere.

The upper threshold was set on the basis of the maximal allowable WSS value inside a scaffold obtained during fluid flow through its pores. It was also determined based on the literature of the reference and set equal to 100 mPa [52]. Higher values can cause call detachment of scaffold walls, which disturbs appropriate cell growth. The results of the obtained WSS are compressively presented in the form of a graph in Figure 11, comparing maximal stresses obtained in the scaffolds of selected pore geometries and porosities. Due to the significant number of obtained stress maps, they are presented in Figures S6–S15 of supplementary materials in order to not disrupt the flow of the main text.

### 3.2. The Influence of Pore Geometry and Scaffold Porosity on the Diffusion of Osteogenic Cells into the Scaffold Pores

**Figure 12:**
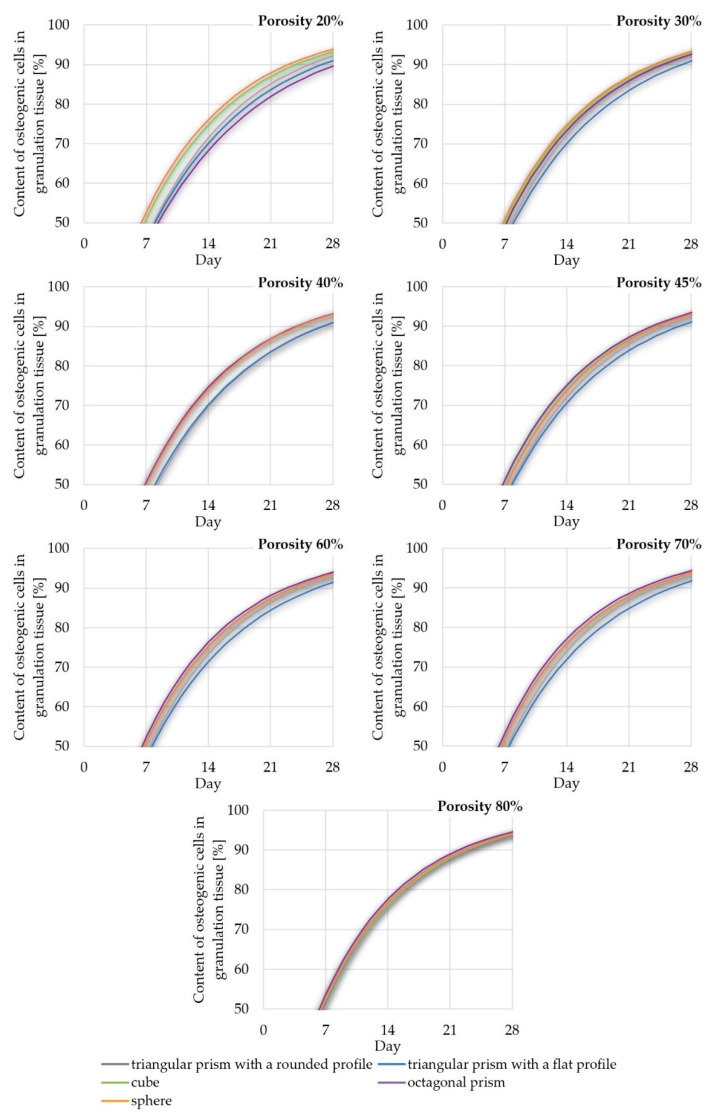
The influence of scaffold geometry on the content of osteogenic cells in granulation tissue within 28 of the healing process.

**Figure 13:**
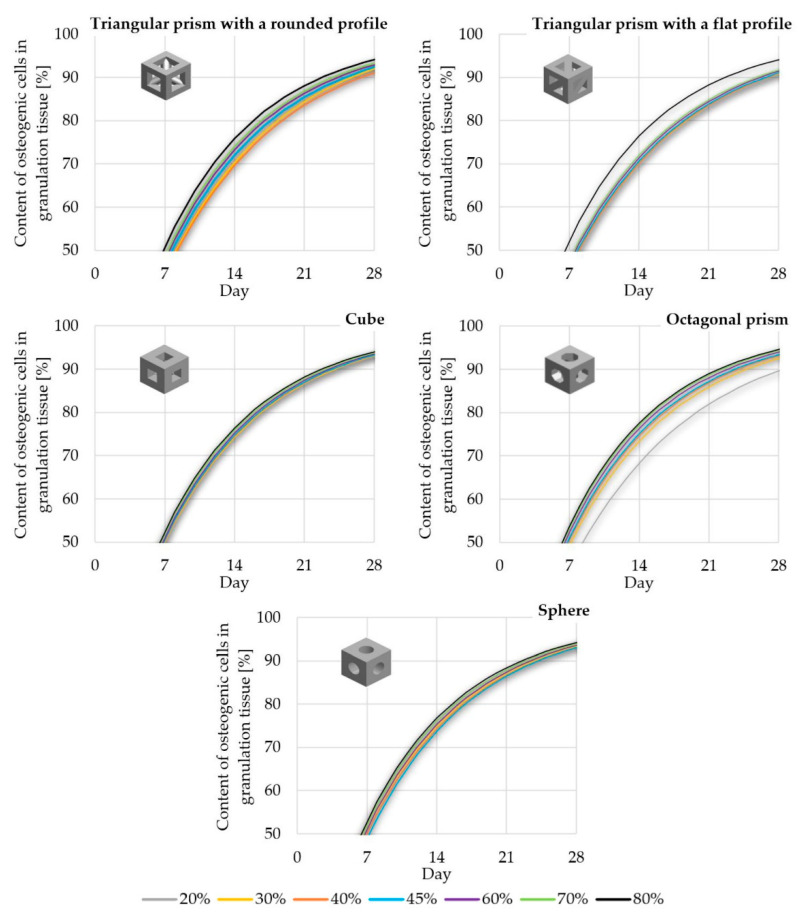
The influence of scaffold porosity on the content of osteogenic cells in granulation tissue within 28 of the healing process.

**Figure 14:**
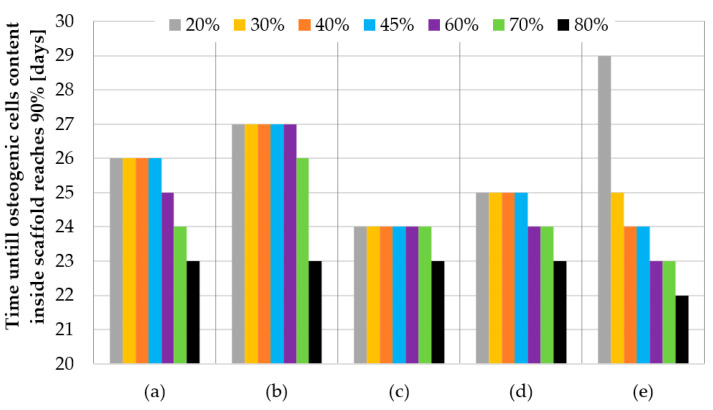
Time needed to fulfil the scaffold with osteogenic cells at 90%: (**a**) triangular prism with a rounded profile, (**b**) triangular prism with a flat profile, (**c**) cube, (**d**) octagonal prism, and (**e**) sphere.

For the sake of clarity, the data obtained was compiled in two comparisons. Figure 12 shows the influence of pore geometry on the percentage of osteogenic cells in granulation tissue, whereas Figure 13 presents the influence of scaffold porosity on the same phenomenon. Figure 12 shows the influence of the scaffold porosity on the studied phenomenon, whereas Figure 13 shows the influence of pore shapes. The simulations were carried out for 50 days after the start of the healing process, but to increase the readability of the results, it was decided to present the obtained data for 28 days, because at that time, for most scaffolds, the cell content was already over 90%. Moreover, the graphs present changes in the percentage of osteogenic cells in a range from 50% to 100%, as under 50% there were no noticeable differences between the results. To increase the readability of the results, an additional figure was included (Figure 14) that presents the time needed to fulfil the scaffold with osteogenic cells at 90%. The healing process was analyzed only in terms of osteogenic cell diffusion. In further research, tissue differentiation within pores can also be included by the use of appropriate mechanoregulation principles and methods. This is especially important in the evaluation of long-term efficiency and secondary osteointegration of orthopedic scaffolds [40]. Due to the significant number of maps showing the process of the propagation of osteogenic cells in granulation tissue, they are presented in the Figures S16–S20 of supplementary materials, in order to not disrupt the flow of the main text.

## 4. Discussion

### 4.1. Efficiency of Pore Geometry and Scaffold Porosity in Terms of Cell Culture Process Conducted in Perfusion Bioreactor

While conducting the overall analyses of all the obtained results, it could be noted that growth medium flowed steadily with a velocity of approximately 0.005 m/s (0–25 mm of a path) before reaching the location of a scaffold (25–35 mm of a path). Then the fluid was pressed into pores of the analyzed structures, which caused a sudden increase of its velocity, which dropped fast when reaching internal part of the pore. This process repeated while pressing the fluid from pore to pore until the end of the scaffold, where the velocity reached again the initial value of approximately 0.005 m/s (35–60 mm of a path). The same phenomenon could be noted for all analyzed structures and pore geometries, but at a different intensity. Its highest intensity was achieved with the use of a triangular prism with a rounded profile as a pore geometry, whereas the lowest was in the case of spherical pores.

There was also another noticeable dependency—the smaller the porosity, the higher the fluid velocity values achieved inside the scaffolds. Moreover, with the increase of porosity, the differences between the maximal and minimal growth medium velocity values obtained inside pores became smaller. The highest flow fluctuations were obtained in the scaffolds with triangular prisms, while other analyzed structural fluctuations were comparable. On this basis it could be concluded that after achieving a certain level of porosity (described in detail in the following bulleted list) that was different for the analyzed geometries, appropriate nutrient exchange between cell cultures inside a scaffold and growth medium may be disturbed. Moreover, at the same time, sufficient gas exchange would also be handicapped. In the end, this could result in creating a non-uniform distribution of developing cells, as mentioned in the data of the reference [12,53,54]. Scaffolds that did not meet the first considered criterium (min. velocity of growth medium inside pores) were:

- Scaffolds with a pore geometry of a triangular prism with a rounded profile and a porosity equal to or higher than 60%;
- Scaffolds with a pore geometry of a triangular prism with a flat profile and a porosity equal to or higher than 30%;
- Scaffolds with a pore geometry of a cube and a porosity equal to or higher than 45%;
- Scaffolds with a pore geometry of an octagonal prism and a porosity equal to or higher than 60%; and
- Scaffolds with a pore geometry of a sphere and a porosity equal to or higher than 45%.

In some cases, the growth medium minimal velocity was not maintained during the flow through a scaffold. In such cases, there were noticeable areas where the growth medium velocity was lower than the required. This velocity vastly increased when fluid was being pressed to the next pore and decreased right after. This may also suggest that in those cases, metabolic waste would not be appropriately removed from the scaffold and the cell culture process would be disturbed [53,54]. From the analyzed structures, only a few were distinguished that fulfilled this requirement:

- Scaffolds with a pore geometry of a cube and a porosity equal to or lower than 30%;
- Scaffolds with a pore geometry of an octagonal prism and a porosity equal to or lower than 30%; and
- Scaffolds with a pore geometry of a sphere and a porosity equal to 20%.

In all the presented models, the highest stresses were obtained in the center of the implants, and the lowest on the outer walls (for detailed insight see Figures S6–S15 in supplementary materials). What is more, the higher the porosity, the lower WSS values obtained. This phenomenon must be taken into consideration while designing the cell culture process individually for the considered internal geometry of a scaffold, as a too-high WSS leads to cell detachment. In extreme cases, WSS can even lead to cell death [55,56]. However, appropriate intensity and distribution of WSS must be maintained, as it stimulates cells to produce extracellular matrix [56]. Scaffolds that did not meet the second considered criterium (max. WSS) were:

- Scaffolds with a pore geometry of a triangular prism with a rounded profile and a porosity equal to or lower than 40%;
- Scaffolds with a pore geometry of a triangular prism with a flat profile and a porosity equal to or lower than 30%;
- Scaffolds with a pore geometry of a cube and a porosity equal to 20%; and
- Scaffolds with a pore geometry of an octagonal prism and a porosity equal to 20%.

All the analyzed types of scaffold with spherical pores and every evaluated porosity fulfilled the second criterium.

Considering both criteria (min. velocity of growth medium inside pores and max. WSS), which could ensure appropriate cell culture process in orthopedic scaffolds inside a perfusion bioreactor (in terms of allowing for appropriate nutrient exchange and cell stimulation), there were single structures that met the assumed requirements, such as:

- Scaffolds with a pore geometry of a cube and a porosity equal to 30%;
- Scaffolds with a pore geometry of an octagonal prism and a porosity equal to 30%; and
- Scaffolds with a pore geometry of a sphere and a porosity equal to 20%.

All findings are summarized in Table 3, which presents the criteria that were fulfilled or failed for each analyzed scaffold geometry and porosity.

### 4.2. Efficiency of Pore Geometry and Scaffold Porosity in Terms of Osteogenic Cell Diffusion after Implantation of a Scaffold

The obtained data suggest that osteogenic cells nearly completely fill granulation tissue within scaffolds in 28 days after initiating the healing process. This is especially important, as they are required for further bridging of bone segments. What is more, appropriate bone healing occurs only when the appropriate content of osteogenic cells is obtained within the callus, as presented by Lacroix et al. [41]. The diffusion starts from the sources of the cells, that is, bone marrow and periosteum (for detailed insight see Figures S16–S20 in supplementary materials). However, the intensity of this phenomenon can be influenced by the analyzed factors of pore geometry and scaffold porosity. Moreover, the higher the porosity, the faster the diffusion process occurs. This phenomenon can also be observed in the data of the reference, which suggest the correctness of the obtained results [39].

Pore geometry had a noticeable impact in the case of lower porosities but only for three out of five analyzed cases. In the case of cubical and spherical pores, the osteogenic cell diffusion efficiency was similar for all considered scaffold porosities. Spherical pore geometry allowed for the highest content of osteogenic cells in granulation tissue to be obtained for porosities equal to or lower than 30%. The pores of the octagonal prism had the lowest efficiency in the porosity case of 20%, but with a porosity equal to or higher than 40%. This pore geometry allowed the highest content of osteogenic cells to be obtained. The differences in efficiency between pore geometries were the highest with the use of a porosity of 20% and decreased with the increase of porosity up to 80%, where no significant differences were noted. The lowest efficiency for most of the analyzed porosities (30–70%) had the pores of a triangular prism with a flat profile.

## 5. Conclusions

The first part of the study allowed for the estimation of growth medium flow through scaffolds of different porosities and pore geometry, whereas the second part approximated the osteogenic cell diffusion process into granulation tissue within scaffolds. The presented comprehensive results allowed for the appropriate selection of scaffold properties to obtain the highest possible functionality in terms of its use in orthopedic regenerative medicine. All of the presented scaffolds can be manufactured with the currently known support-free 3D-printing methods, such as selective laser sintering (SLS), which makes it possible to use them in experimental conditions. The data presented can also be a starting point for further analyses in order to increase the quality of the analyzed structures and in the end to increase the efficiency of regenerative medicine.

## Figures and Tables

**Table 1:** Values of parameters used in the study to evaluate the growth medium velocity and wall shear stress inside the pores of analyzed scaffolds.

Parameter	Value	Reference
Density [kg/m^3^]	1040	[28]
Dynamic viscosity [Pa·s]	0.00081	[29]
Initial velocity [m/s]	0.0005	[29]
Temperature [K]	310.15	[29]

**Table 2:** Material properties used in the study to evaluate the influence of pore geometry and scaffold porosity on osteogenic cell diffusion in granulation tissue.

Tissue Type/Implant Type	Density [kg/cm^3^]	Poisson’s Ratio	Young’s Modulus [GPa]	Diffusion Coefficient [m^2^/Day]	Reference
Cortical bone	1740	0.30	20	-	[49]
Bone marrow	1100	0.17	0.002	-	[50]
Granulation tissue	910	0.17	0.0001	2.5	[50]
Scaffold (Ti6Al4V)	4500	0.33	110	-	[51]

**Table 3:** Summary of the efficiency of pore geometry and scaffold porosity in terms of the selected criteria.

Scaffold Feature			Scaffold Geometry						
			(a)	(b)		(c)		(d)	(e)
Scaffold Porosity		20%	−	−		+		+	+
			−	−		−		−	+
		30%	−	−		+		+	−
			−	−		+		+	+
		40%	−	−		−		−	−
			−	+		+		+	+
		45%	−	−		−		−	−
			+	+		+		+	+
		60%	−	−		−		−	−
			+	+		+		+	+
		70%	−	−		−		−	−
			+	+		+		+	+
		80%	−	−		−		−	−
			+	+		+		+	+
	Min. Velocity Criterium						Max. WSS Criterium		
	
+	Both Criteria Fulfilled				−		Both Criteria Failed		
+					−				
−	Upper Criteria Failed/Lower Fulfilled				+		Upper Criteria Fulfilled/Lower Failed		
+					−				

Note: (**a**) triangular prism with a rounded profile, (**b**) triangular prism with a flat profile, (**c**) cube, (**d**) octagonal prism, and (**e**) sphere.

## Data Availability

Data is contained within the article or supplementary materials.

## Supplementary Materials

The following are available online at https://www.mdpi.com/1996-1944/14/1/109/s1, Figure S1: Streamlines of growth medium velocity in a scaffold with pores geometry of triangular prism with a rounded profile and various porosity: (a) 20% porosity; (b) 30% porosity; (c) 40% porosity; (d) 45% porosity; (e) 60% porosity; (f) 70% porosity; (g) 80% porosity; Figure S2: Streamlines of growth medium velocity in a scaffold with pores geometry of triangular prism with a flat profile and various porosity: (a) 20% porosity; (b) 30% porosity; (c) 40% porosity; (d) 45% porosity; (e) 60% porosity; (f) 70% porosity; (g) 80% porosity; Figure S3: Streamlines of growth medium velocity in a scaffold with pores geometry of cube and various porosity: (a) 20% porosity; (b) 30% porosity; (c) 40% porosity; (d) 45% porosity; (e) 60% porosity; (f) 70% porosity; (g) 80% porosity; Figure S4: Streamlines of growth medium velocity in a scaffold with pores geometry of octagonal prism and various porosity: (a) 20% porosity; (b) 30% porosity; (c) 40% porosity; (d) 45% porosity; (e) 60% porosity; (f) 70% porosity; (g) 80% porosity; Figure S5: Streamlines of growth medium velocity in a scaffold with pores geometry of sphere and various porosity: (a) 20% porosity; (b) 30% porosity; (c) 40% porosity; (d) 45% porosity; (e) 60% porosity; (f) 70% porosity; (g) 80% porosity; Figure S6: WSS distribution in isometric and cross-section views in a scaffold with pores geometry of triangular prism with a rounded profile and various porosity: (a) 20% porosity; (b) 30% porosity; (c) 40% porosity; (d) 45% porosity; (e) 60% porosity; (f) 70% porosity; (g) 80% porosity; Figure S7: WSS distribution in isometric double cross-section view in a scaffold with pores geometry of triangular prism with a rounded profile and various porosity: (a) 20% porosity; (b) 30% porosity; (c) 40% porosity; (d) 45% porosity; (e) 60% porosity; (f) 70% porosity; (g) 80% porosity; Figure S8: WSS distribution in isometric and cross-section views in a scaffold with pores geometry of triangular prism with a flat profile and various porosity: (a) 20% porosity; (b) 30% porosity; (c) 40% porosity; (d) 45% porosity; (e) 60% porosity; (f) 70% porosity; (g) 80% porosity; Figure S9: WSS distribution in isometric double cross-section view in a scaffold with pores geometry of triangular prism with a flat profile and various porosity: (a) 20% porosity; (b) 30% porosity; (c) 40% porosity; (d) 45% porosity; (e) 60% porosity; (f) 70% porosity; (g) 80% porosity; Figure S10: WSS distribution in isometric and cross-section views in a scaffold with pores geometry of cube and various porosity: (a) 20% porosity; (b) 30% porosity; (c) 40% porosity; (d) 45% porosity; (e) 60% porosity; (f) 70% porosity; (g) 80% porosity; Figure S11: WSS distribution in isometric double cross-section view in a scaffold with pores geometry of cube and various porosity: (a) 20% porosity; (b) 30% porosity; (c) 40% porosity; (d) 45% porosity; (e) 60% porosity; (f) 70% porosity; (g) 80% porosity; Figure S12: WSS distribution in isometric and cross-section views in a scaffold with pores geometry of octagonal prism and various porosity: (a) 20% porosity; (b) 30% porosity; (c) 40% porosity; (d) 45% porosity; (e) 60% porosity; (f) 70% porosity; (g) 80% porosity; Figure S13: WSS distribution in isometric double cross-section view in a scaffold with pores geometry of octagonal prism and various porosity: (a) 20% porosity; (b) 30% porosity; (c) 40% porosity; (d) 45% porosity; (e) 60% porosity; (f) 70% porosity; (g) 80% porosity; Figure S14: WSS distribution in isometric and cross-section views in a scaffold with pores geometry of sphere and various porosity: (a) 20% porosity; (b) 30% porosity; (c) 40% porosity; (d) 45% porosity; (e) 60% porosity; (f) 70% porosity; (g) 80% porosity; Figure S15: WSS distribution in isometric double cross-section view in a scaffold with pores geometry of sphere and various porosity: (a) 20% porosity; (b) 30% porosity; (c) 40% porosity; (d) 45% porosity; (e) 60% porosity; (f) 70% porosity; (g) 80% porosity; Figure S16: Osteogenic cells diffusion intensity in granulation tissue inside scaffold with pores geometry of triangular prism with a rounded profile and various porosity; Figure S17: Osteogenic cells diffusion intensity in granulation tissue inside scaffold with pores geometry of triangular prism with a flat profile and various porosity; Figure S18: Osteogenic cells diffusion intensity in granulation tissue inside scaffold with pores geometry of cube and various porosity; Figure S19: Osteogenic cells diffusion intensity in granulation tissue inside scaffold with pores geometry of octagonal prism and various porosity; Figure S20: Osteogenic cells diffusion intensity in granulation tissue inside scaffold with pores geometry of sphere and various porosity.
