# Supplementary material for: Numerical Analysis of the Influence of Porosity and Pore Geometry on Functionality of Scaffolds Designated for Orthopedic Regenerative Medicine
Source: Materials (Basel). 2020 Dec 29;14(1):109. doi: 10.3390/ma14010109 (PMC7796183; doi:10.3390/ma14010109)
Supplement: Supplementary file 1 [file materials-14-00109-s001.pdf]

materials

MDPI

Supplementary Materials

Numerical Analysis of the Influence of Porosity and Pore Geometry on Functionality of Scaffolds Designated for Orthopedic Regenerative Medicine

Piotr Prochor * and Anita Gryko

Institute of Biomedical Engineering, Faculty of Mechanical Engineering, Bialystok University of Technology, Wiejska 45C Street, 15-351 Bialystok, Poland; grykoanita@gmail.com
* Correspondence: p.prochor@pb.edu.pl; Tel.: +48-571-443-048

Citation: Prochor, P.; Gryko, A. Numerical analysis of the influence of porosity and pore geometry on functionality of scaffolds designated for orthopedic regenerative medicine. *Materials* **2021**, *14*, 109. https://doi.org/10.3390/ma14010109

Received: 23 November 2020
Accepted: 24 December 2020
Published: 29 December 2020

Publisher's Note: MDPI stays neutral with regard to jurisdictional claims in published maps and institutional affiliations.

Copyright: © 2020 by the authors. Licensee MDPI, Basel, Switzerland. This article is an open access article distributed under the terms and conditions of the Creative Commons Attribution (CC BY) license (http://creativecommons.org/licenses/by/4.0/).

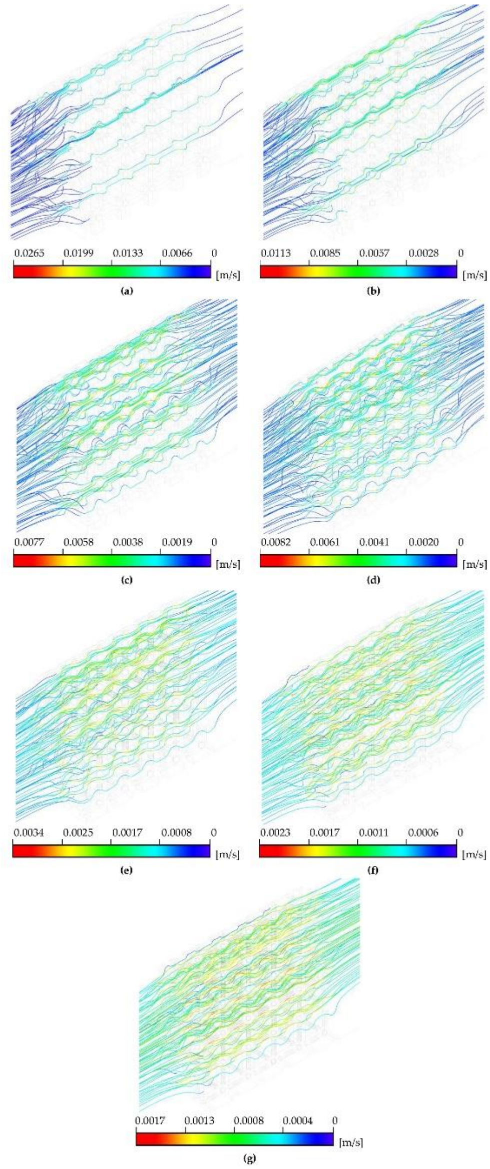

Figure S1. Streamlines of growth medium velocity in a scaffold with pores geometry of triangular prism with a rounded profile and various porosity: (**a**) 20% porosity; (**b**) 30% porosity; (**c**) 40% porosity; (**d**) 45% porosity; (**e**) 60% porosity; (**f**) 70% porosity; (**g**) 80% porosity.

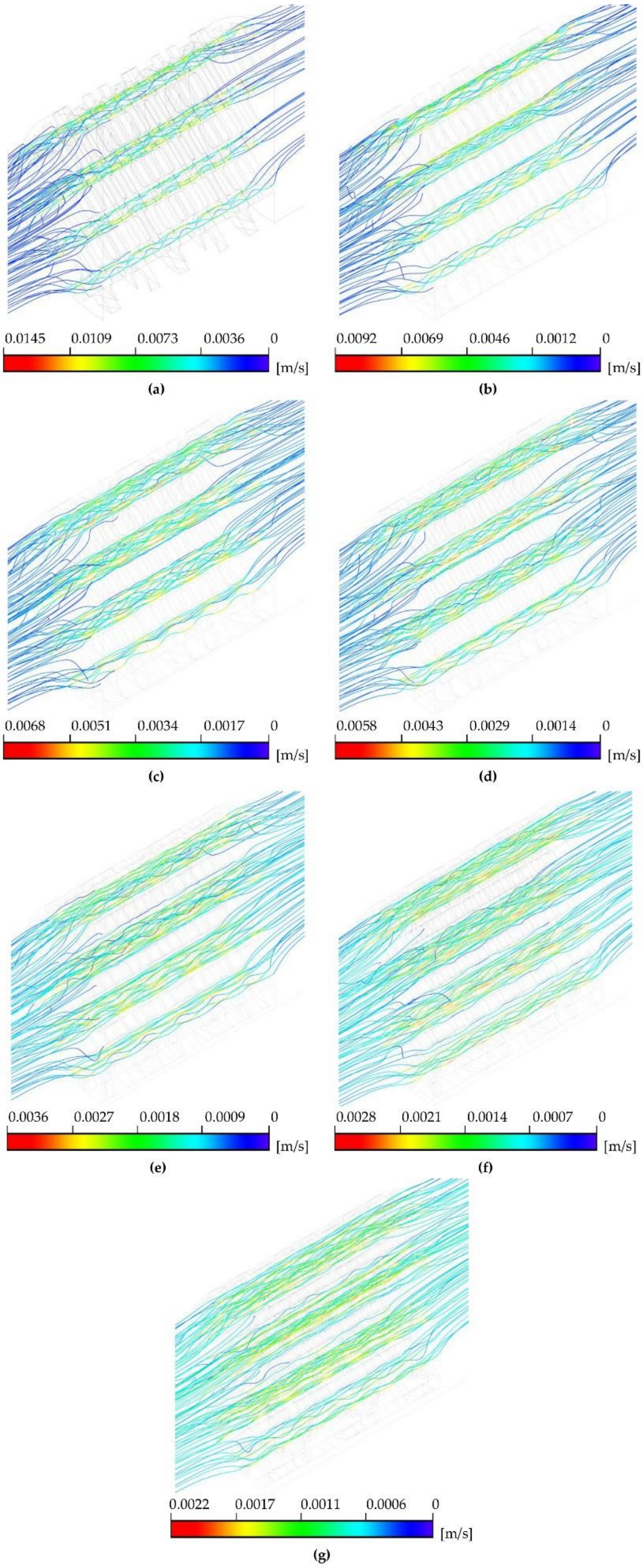

Figure S2. Streamlines of growth medium velocity in a scaffold with pores geometry of triangular prism with a flat profile and various porosity: (**a**) 20% porosity; (**b**) 30% porosity; (**c**) 40% porosity; (**d**) 45% porosity; (**e**) 60% porosity; (**f**) 70% porosity; (**g**) 80% porosity.

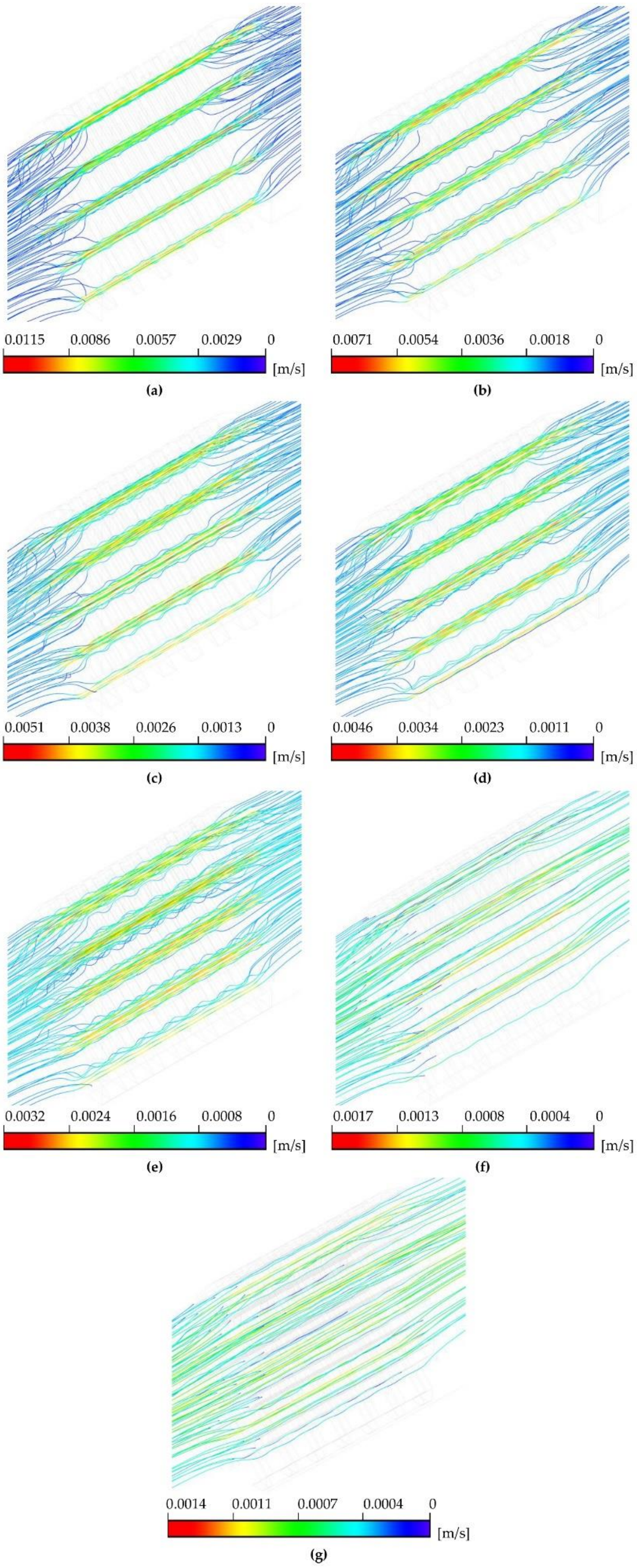

Figure S3. Streamlines of growth medium velocity in a scaffold with pores geometry of cube and various porosity: (**a**) 20% porosity; (**b**) 30% porosity; (**c**) 40% porosity; (**d**) 45% porosity; (**e**) 60% porosity; (**f**) 70% porosity; (**g**) 80% porosity.

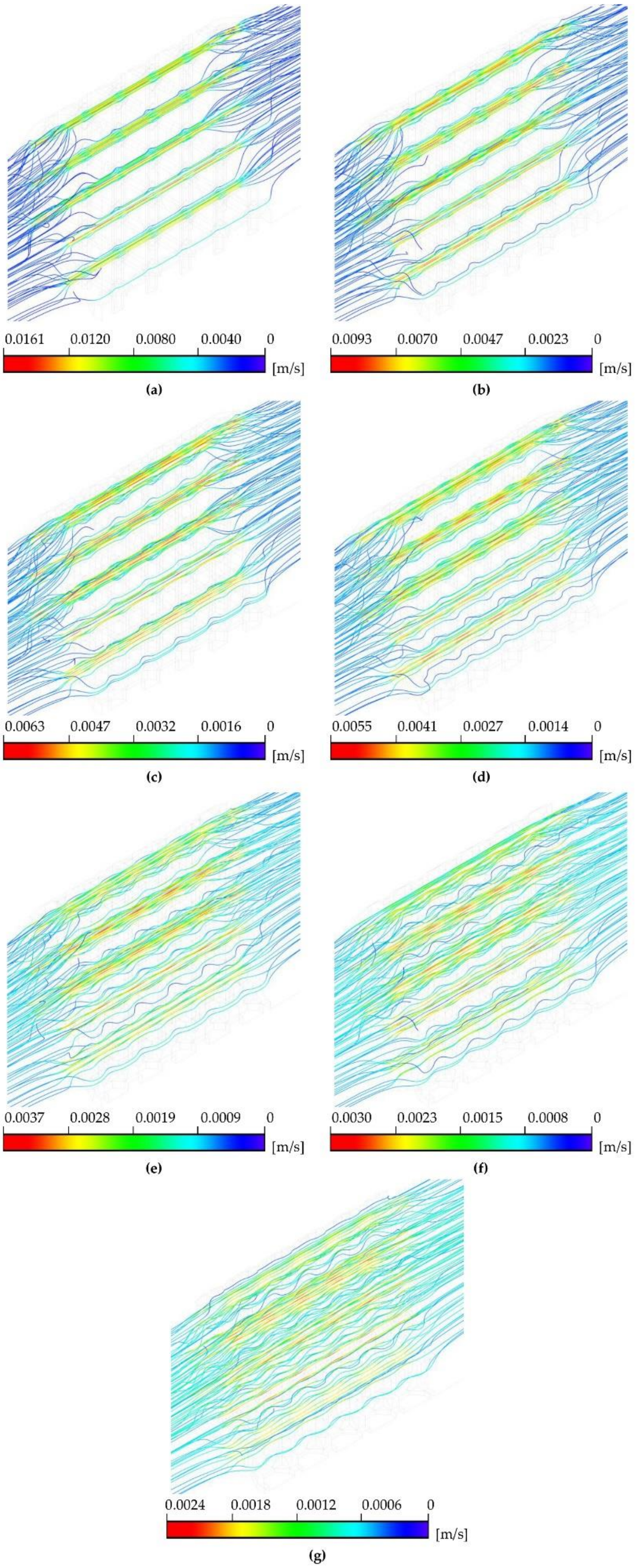

Figure S4. Streamlines of growth medium velocity in a scaffold with pores geometry of octagonal prism and various porosity: (**a**) 20% porosity; (**b**) 30% porosity; (**c**) 40% porosity; (**d**) 45% porosity; (**e**) 60% porosity; (**f**) 70% porosity; (**g**) 80% porosity.

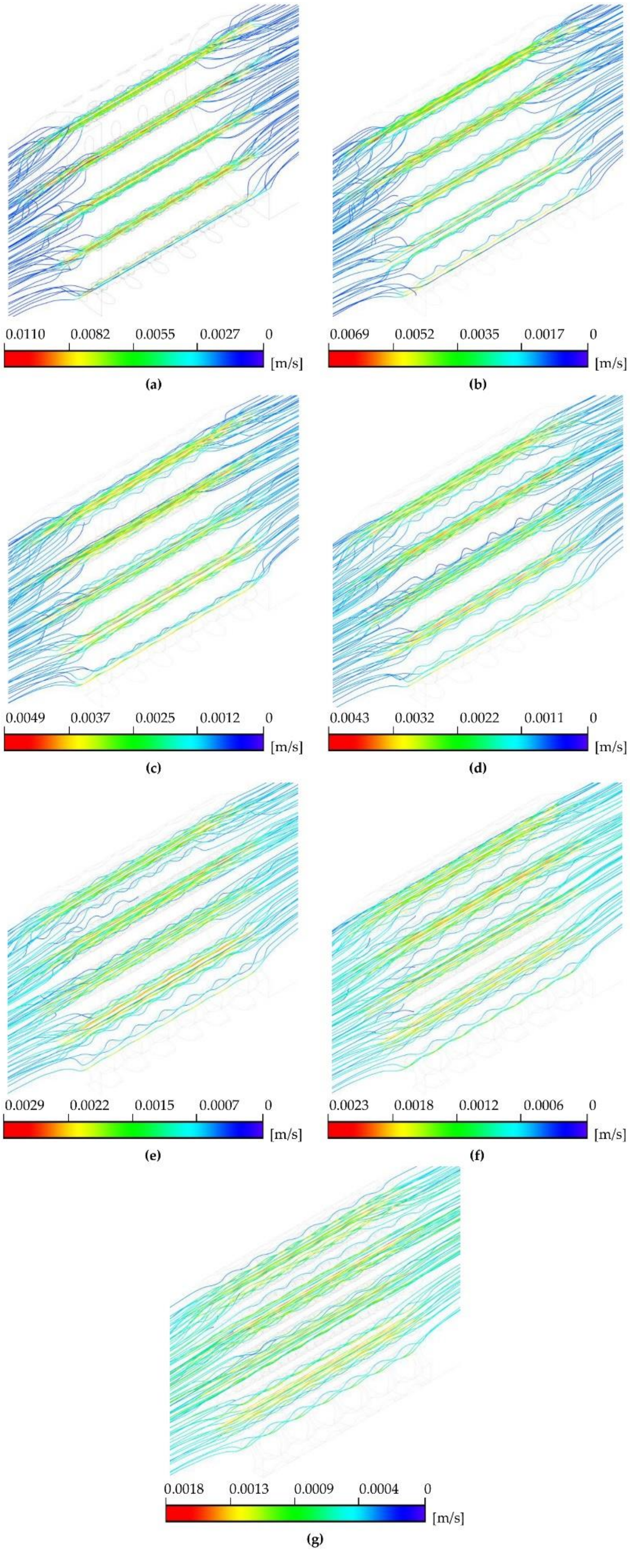

Figure S5. Streamlines of growth medium velocity in a scaffold with pores geometry of sphere and various porosity: (**a**) 20% porosity; (**b**) 30% porosity; (**c**) 40% porosity; (**d**) 45% porosity; (**e**) 60% porosity; (**f**) 70% porosity; (**g**) 80% porosity.

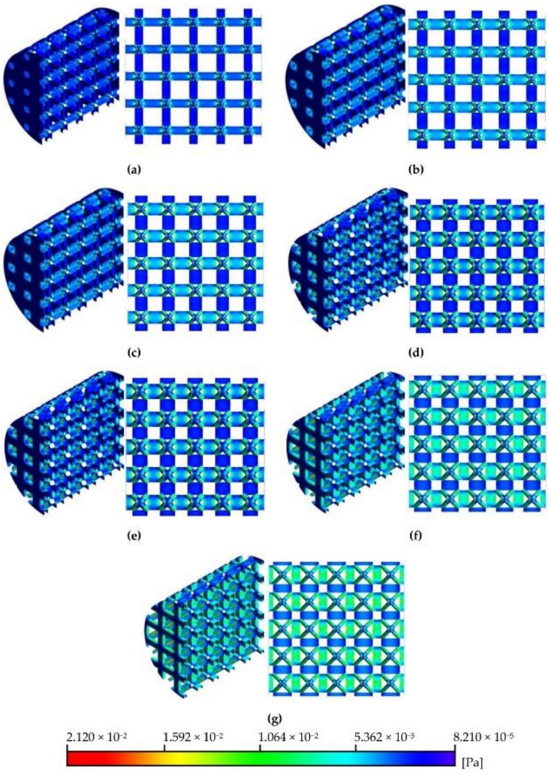

Figure S6. WSS distribution in isometric and cross-section views in a scaffold with pores geometry of triangular prism with a rounded profile and various porosity: (**a**) 20% porosity; (**b**) 30% porosity; (**c**) 40% porosity; (**d**) 45% porosity; (**e**) 60% porosity; (**f**) 70% porosity; (**g**) 80% porosity.

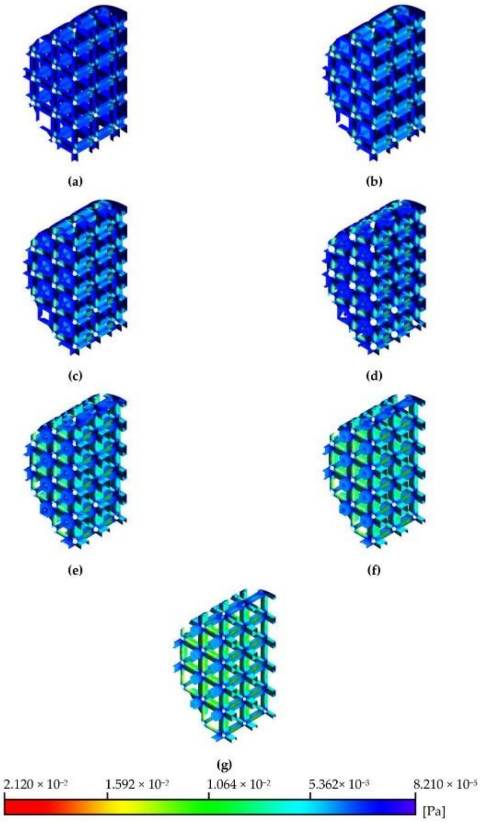

Figure S7. WSS distribution in isometric double cross-section view in a scaffold with pores geometry of triangular prism with a rounded profile and various porosity: (**a**) 20% porosity; (**b**) 30% porosity; (**c**) 40% porosity; (**d**) 45% porosity; (**e**) 60% porosity; (**f**) 70% porosity; (**g**) 80% porosity.

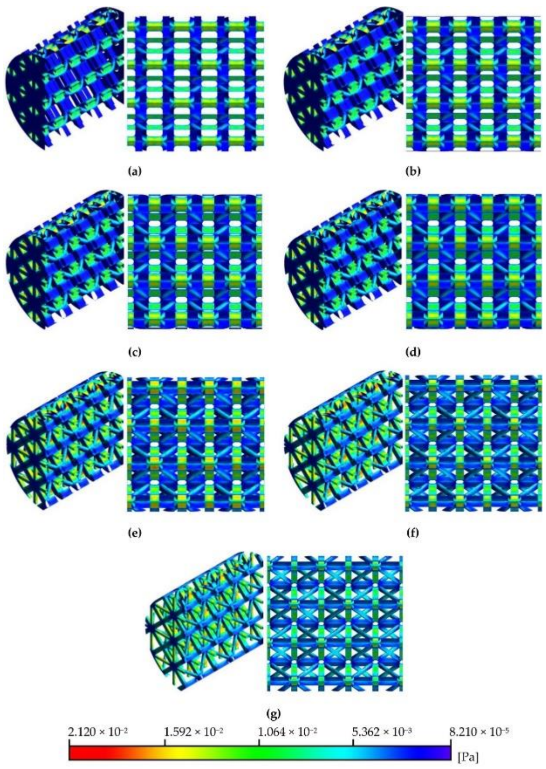

Figure S8. WSS distribution in isometric and cross-section views in a scaffold with pores geometry of triangular prism with a flat profile and various porosity: (**a**) 20% porosity; (**b**) 30% porosity; (**c**) 40% porosity; (**d**) 45% porosity; (**e**) 60% porosity; (**f**) 70% porosity; (**g**) 80% porosity.

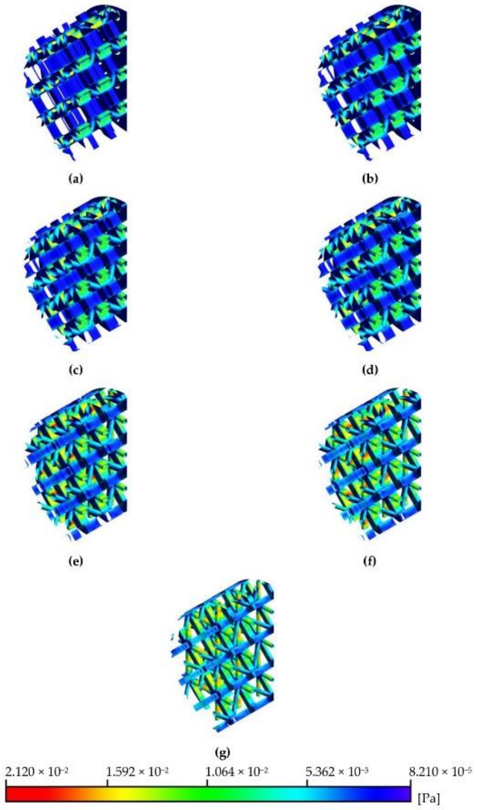

Figure S9. WSS distribution in isometric double cross-section view in a scaffold with pores geometry of triangular prism with a flat profile and various porosity: (**a**) 20% porosity; (**b**) 30% porosity; (**c**) 40% porosity; (**d**) 45% porosity; (**e**) 60% porosity; (**f**) 70% porosity; (**g**) 80% porosity.

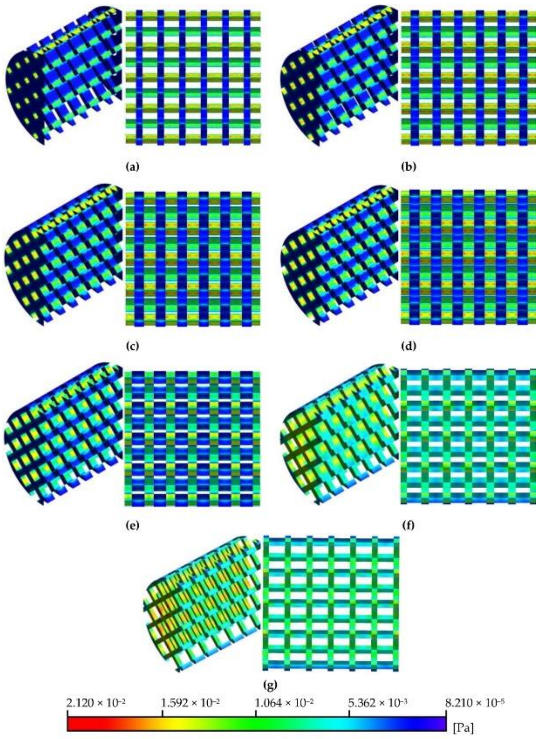

Figure S10. WSS distribution in isometric and cross-section views in a scaffold with pores geometry of cube and various porosity: (**a**) 20% porosity; (**b**) 30% porosity; (**c**) 40% porosity; (**d**) 45% porosity; (**e**) 60% porosity; (**f**) 70% porosity; (**g**) 80% porosity.

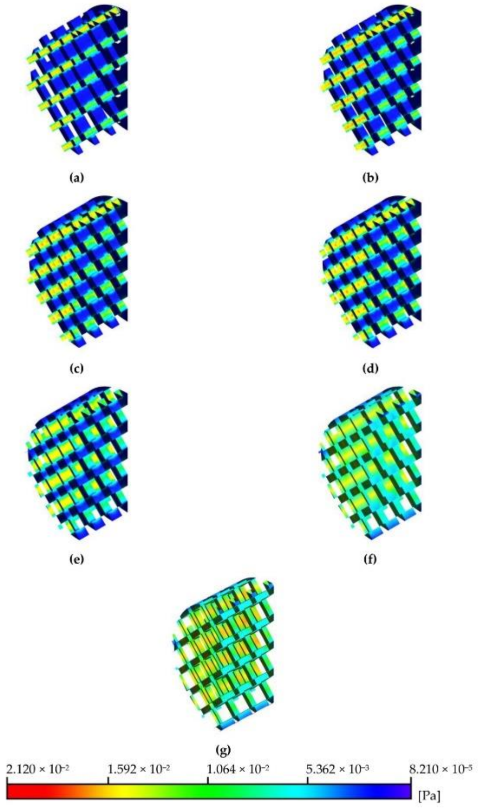

Figure S11. WSS distribution in isometric double cross-section view in a scaffold with pores geometry of cube and various porosity: (**a**) 20% porosity; (**b**) 30% porosity; (**c**) 40% porosity; (**d**) 45% porosity; (**e**) 60% porosity; (**f**) 70% porosity; (**g**) 80% porosity.

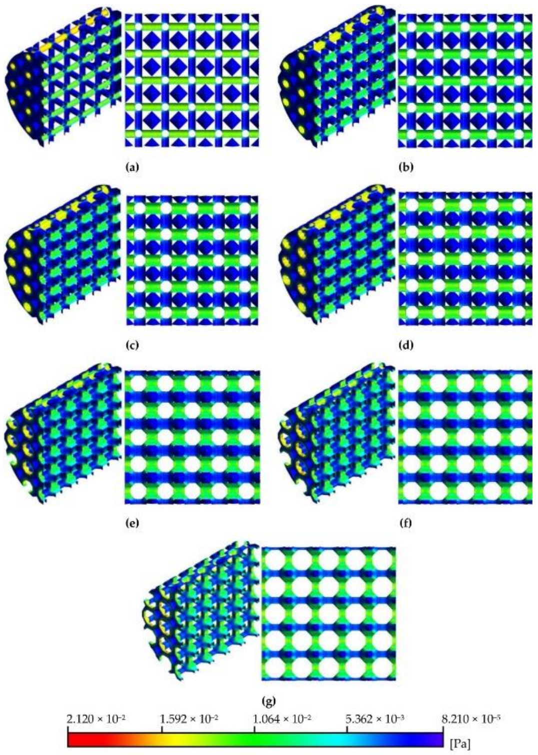

Figure S12. WSS distribution in isometric and cross-section views in a scaffold with pores geometry of octagonal prism and various porosity: (**a**) 20% porosity; (**b**) 30% porosity; (**c**) 40% porosity; (**d**) 45% porosity; (**e**) 60% porosity; (**f**) 70% porosity; (**g**) 80% porosity.

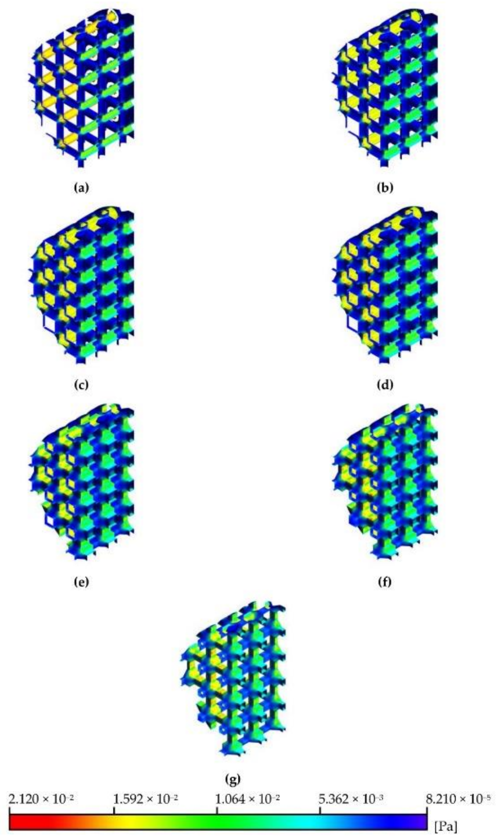

Figure S13. WSS distribution in isometric double cross-section view in a scaffold with pores geometry of octagonal prism and various porosity: (**a**) 20% porosity; (**b**) 30% porosity; (**c**) 40% porosity; (**d**) 45% porosity; (**e**) 60% porosity; (**f**) 70% porosity; (**g**) 80% porosity.

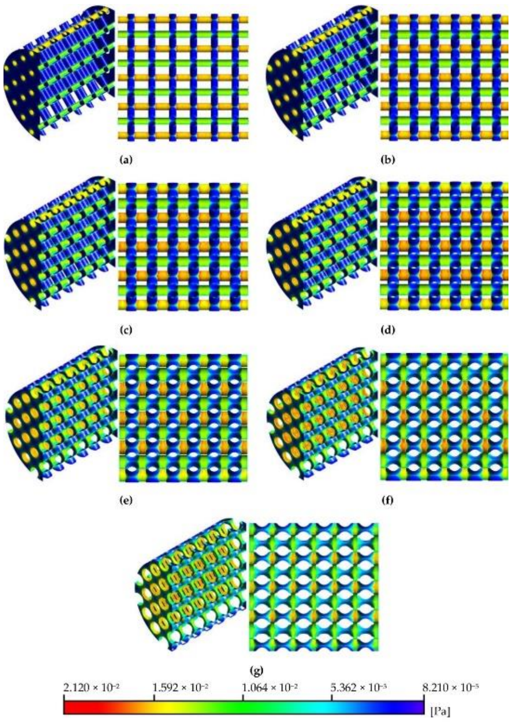

Figure S14. WSS distribution in isometric and cross-section views in a scaffold with pores geometry of sphere and various porosity: (**a**) 20% porosity; (**b**) 30% porosity; (**c**) 40% porosity; (**d**) 45% porosity; (**e**) 60% porosity; (**f**) 70% porosity; (**g**) 80% porosity.

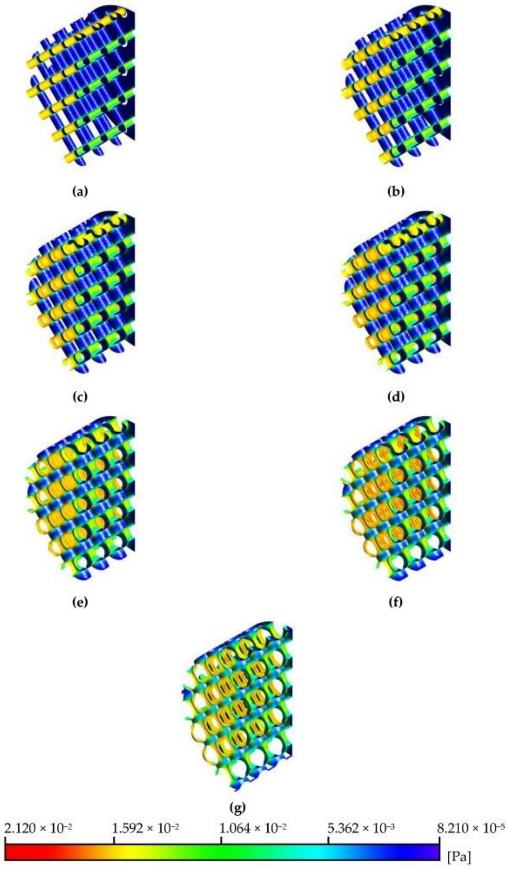

Figure S15. WSS distribution in isometric double cross-section view in a scaffold with pores geometry of sphere and various porosity: (**a**) 20% porosity; (**b**) 30% porosity; (**c**) 40% porosity; (**d**) 45% porosity; (**e**) 60% porosity; (**f**) 70% porosity; (**g**) 80% porosity.

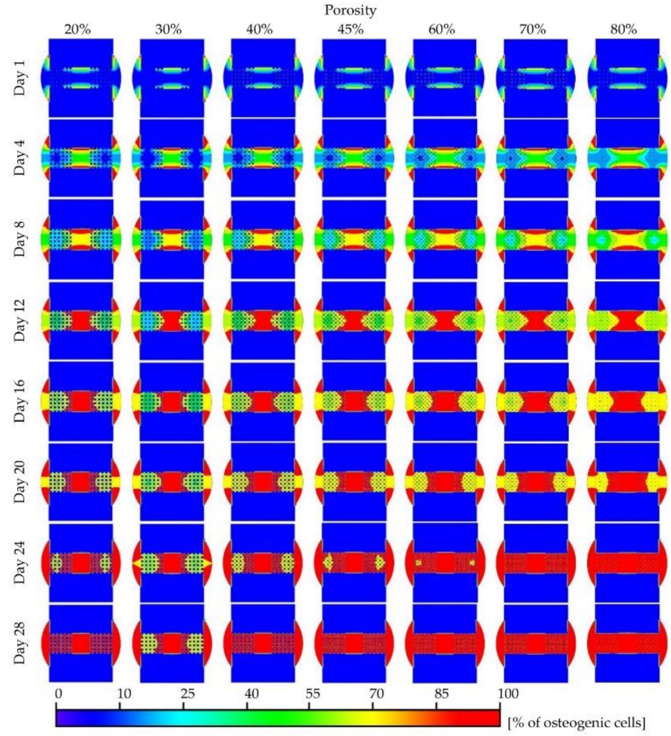

Figure S16. Osteogenic cells diffusion intensity in granulation tissue inside scaffold with pores geometry of triangular prism with a rounded profile and various porosity.

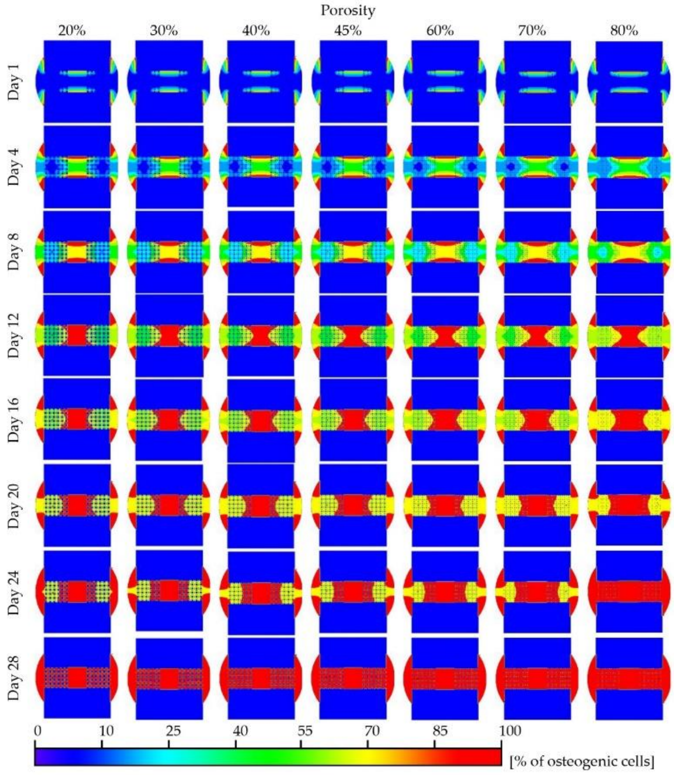

Figure S17. Osteogenic cells diffusion intensity in granulation tissue inside scaffold with pores geometry of triangular prism with a flat profile and various porosity.

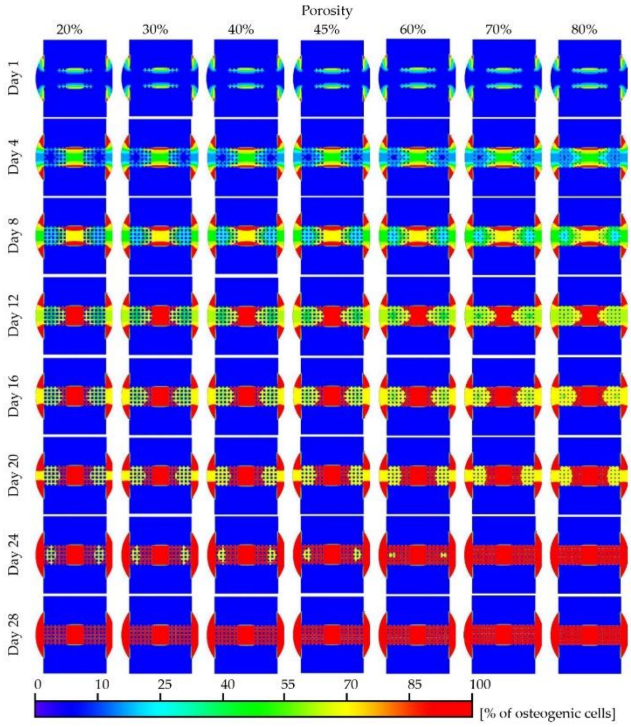

Figure S18. Osteogenic cells diffusion intensity in granulation tissue inside scaffold with pores geometry of cube and various porosity.

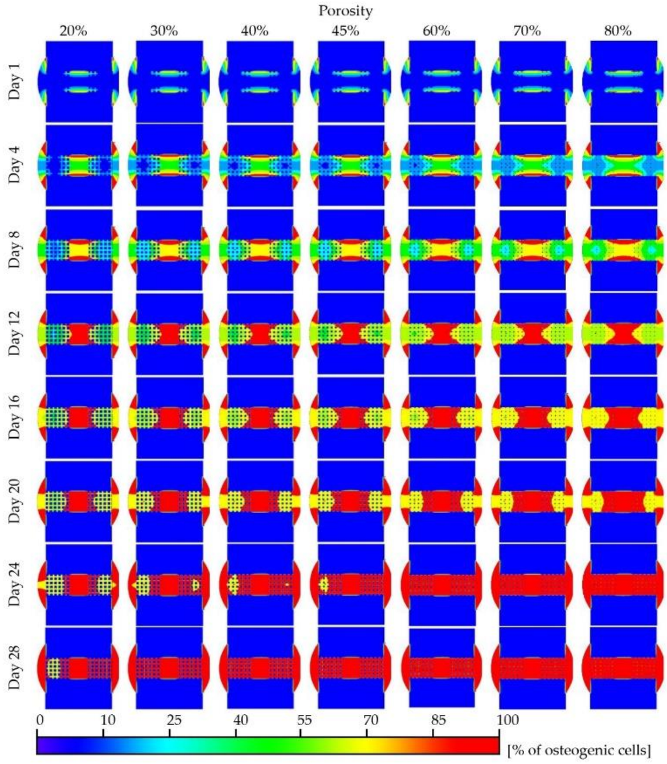

Figure S19. Osteogenic cells diffusion intensity in granulation tissue inside scaffold with pores geometry of octagonal prism and various porosity.

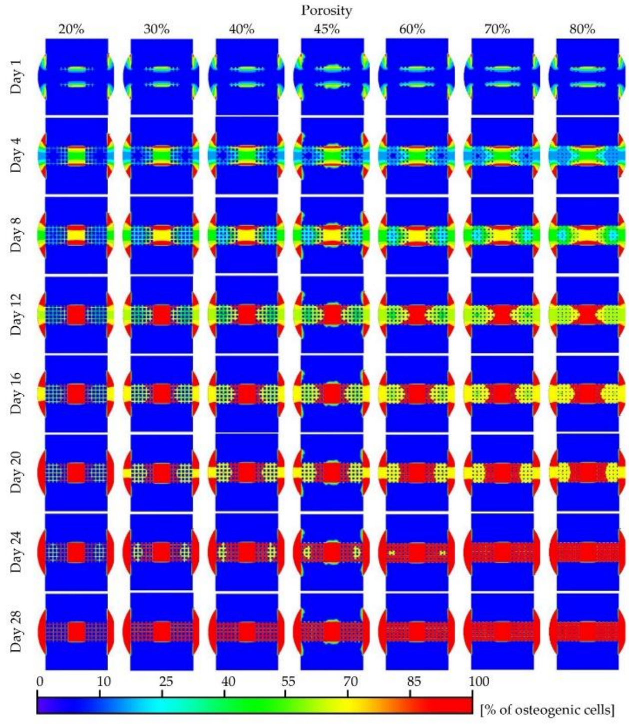

Figure S20. Osteogenic cells diffusion intensity in granulation tissue inside scaffold with pores geometry of sphere and various porosity.
